# Prediction of Ethanol-Mediated Growth Morphology of Ammonium Dinitramide/Pyrazine-1,4-Dioxide Cocrystal at Different Temperatures

**DOI:** 10.3390/molecules28114534

**Published:** 2023-06-03

**Authors:** Yuanping Zhang, Boyu Ma, Xinlei Jia, Conghua Hou

**Affiliations:** 1 School of Coal Engineering, Shanxi Datong University, Datong 037003, China; 18234095862@163.com (Y.Z.); 17835702113@163.com (B.M.); 2 Department of Chemical Engineering and Safety, Binzhou University, Binzhou 256603, China; 3 School of Environment and Safety Engineering, North University of China, Taiyuan 030051, China; houconghua@163.com

**Keywords:** ADN/PDO cocrystal, attachment energy model, molecular dynamics, growth morphology

## Abstract

The crystal morphology of high energetic materials plays a crucial role in aspects of their safety performance such as impact sensitivity. In order to reveal the crystal morphology of ammonium dinitramide/pyrazine-1,4-dioxide (ADN/PDO) cocrystal at different temperatures, the modified attachment energy model (MAE) was used at 298, 303, 308, and 313 K to predict the morphology of the ADN/PDO cocrystal under vacuum and ethanol. The results showed that under vacuum conditions, five growth planes of the ADN/PDO cocrystal were given, which were (1 0 0), (0 1 1), (1 1 0), (1 1 −1), and (2 0 −2). Among them, the ratios of the (1 0 0) and (0 1 1) planes were 40.744% and 26.208%, respectively. In the (0 1 1) crystal plane, the value of S was 1.513. The (0 1 1) crystal plane was more conducive to the adsorption of ethanol molecules. The order of binding energy between the ADN/PDO cocrystal and ethanol solvent was (0 1 1) > (1 1 −1) > (2 0 −2) > (1 1 0) > (1 0 0). The radial distribution function analysis revealed that there were hydrogen bonds between the ethanol and the ADN cations, van der Waals interactions with the ADN anions. As the temperature increased, the aspect ratio of the ADN/PDO cocrystal was reduced, making the crystal more spherical, which helped to further reduce the sensitivity of this explosive.

## 1. Introduction

High-energy compounds are a class of compounds containing explosive groups that can independently undergo chemical reactions and output energy. They are widely used in exploration, aviation, and other fields. Ammonium dinitramide (ADN) [1] is an ionic energetic compound, which is in the α crystal form at normal temperature and pressure, with a crystal density of 1.812 g/cm^3^ [2], an enthalpy of formation of −1.22 mJ/kg [3], and an oxygen balance of 25.8% [4]. Compared with ammonium perchlorate (AP), ADN has a higher enthalpy of formation [5], which can provide higher specific impulse when used in propellants. Since ADN is free of chlorine, its combustion products are clean, and its characteristic signal, such as primary smoke, etc., is low when it burns. However, the application of ADN is limited by the high hygroscopicity at room temperature [6,7].

Cocrystallization of energetic materials can effectively improve the physical and chemical properties [8,9]. Previous studies [10,11] have shown that cocrystallization is an effective way to improve the hygroscopicity of ADN. The moisture absorption rate experiment with an AD/18C6 (18-crown-6) cocrystal [12] showed that the hygroscopicity of the ADN/18C6 cocrystal was significantly lower than that of the ADN. The modified attachment energy was adopted by Xie [13] to explore the influence of ethanol on the morphology of an ADN/18C6 cocrystal at different temperatures. The results revealed that the morphology of the ADN/18C6 cocrystal was close to spherical at 293 K, and the cocrystal of the ADN/18C6 had lower hygroscopicity than the ADN at this temperature. Pyrazine-1,4-dioxide (PDO) was selected by Michael K [14] to construct an ADN/PDO cocrystal with a molar ratio of 2:1. The hygroscopicity test showed that the hygroscopicity of the ADN decreased significantly after the cocrystallization of the ADN and PDO. The thermal stability of the ADN/PDO cocrystal was higher than the ADN. Meanwhile, the energetic properties were improved over the ADN.

As an important indicator of crystals, their morphology has been widely considered [15,16,17]. The morphology of an energetic material can significantly affect its sensitivity [18], which is affected by saturation, solvent, temperature, etc. [19,20]. In this work, an “explosive/solvent” double-layer model was constructed, and four temperatures were adopted to perform the molecular dynamics, which was carried out at 298, 303, 308, and 313 K. The morphology of the ADN/PDO cocrystal was predicted with the help of a modified attachment energy model. The binding energy, radial distribution function, and diffusion coefficient were used to reveal the growth morphology of the ADN/PDO cocrystal in an ethanol environment.

## 2. Results and Discussion

### 2.1. Force Field Verification

**Figure 1:**
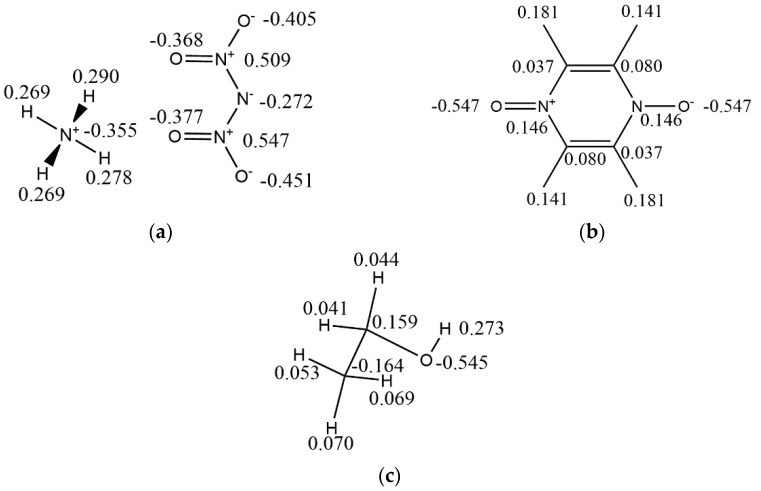
Mulliken charges of the ADN, PDO, and ethanol. (**a**) ADN (**b**) PDO (**c**) EtOH.

As an ionic compound, ADN has strong electrostatic interactions between anions and cations. In order to accurately calculate the interaction energy between the ADN/PDO and ethanol, the DMol^3^ program was selected to calculate the Mulliken charge of the ADN/PDO unitcell and ethanol, which was adopted as the atomic charge for subsequent calculations. The Perdew–Burke–Ernzerhof (PBE) correlation was used to calculate the exchange-correlation energy. The all-electron method was implemented to treat the core electrons. The double numerical plus polarization numerical basis set (DNP) was adapted. The Mulliken charge is shown in Figure 1.

In classical molecular dynamics, the force field parameters directly determine the accuracy of calculation results. In order to accurately describe the interaction in the ADN/PDO cocrystal, COMPASSIII, PCFF, CVFF, Universal, and Dreiding force fields and the Mulliken charge were adopted to describe the properties of the atom in the whole simulation model. The optimized unit cell parameters of the ADN/PDO cocrystal are given in Table 1. The relative error (RE) of the unit cell parameters in Table 1 revealed that the RE optimized by CVFF was smaller than the results under other force fields, and it was closer to the experimental value. Meanwhile, the calculation of Yusop [21] revealed that the CVFF force field was suitable for the molecular dynamics simulation of ethanol. Therefore, the CVFF force field was adopted to perform the molecular dynamics simulation.

### 2.2. Morphology of the ADN/PDO in Vacuum

**Figure 2:**
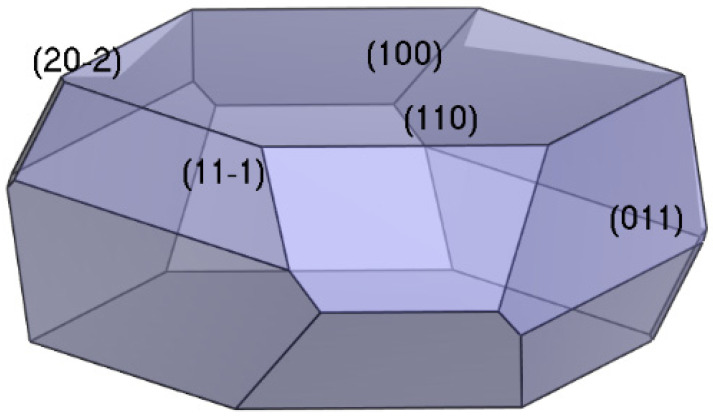
The morphology of the ADN/PDO cocrystal under vacuum (attachment energy model).

The morphology of the ADN/PDO in vacuum was calculated based on the AE model, as shown in Figure 2. Under vacuum conditions, the important growth planes and area ratios of the ADN/PDO cocrystal are shown in Table 2. It can be seen from Figure 2 that the vacuum morphology of the ADN/PDO cocrystal was approximately hexagonal prism, and the aspect ratio of the crystal was 2.569. Its crystal morphology was mainly composed of five growth crystal planes (1 0 0), (0 1 1), (1 1 0), (1 1 −1), and (2 0 −2). Among them, the (1 0 0) surface had the largest exposed area, accounting for 40.744%, and had the greatest morphological importance. According to Formula (3), it can be seen that the absolute value of the attachment energy was proportional to the relative growth rate of the corresponding crystal plane. The order of the relative growth rate of each crystal plane of the ADN/PDO was (2 0 −2) > (1 1 −1) > (1 1 0) > (0 1 1) > (1 0 0).

**Figure 3:**
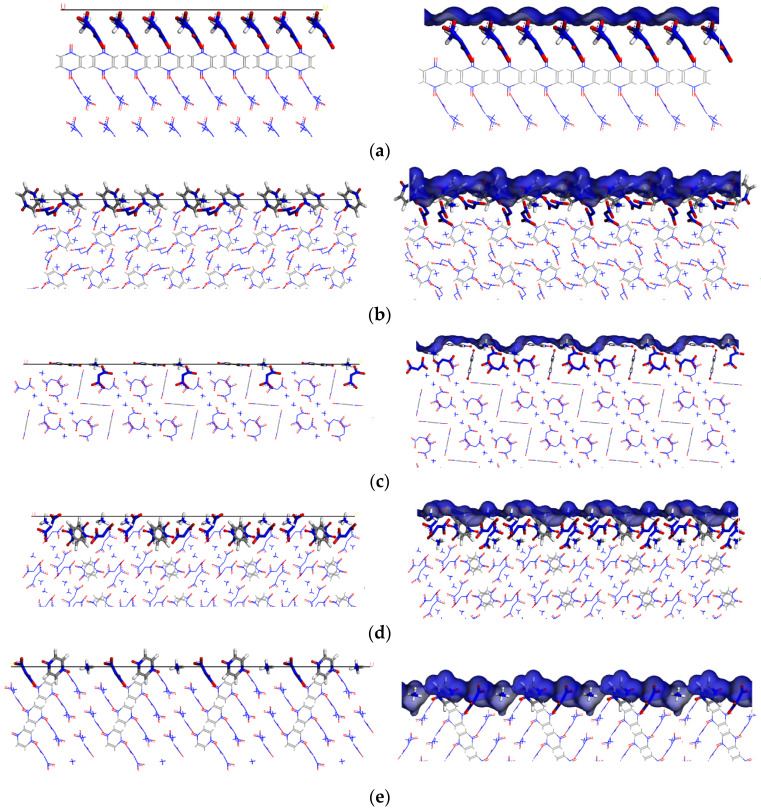
The molecular stacking structure (**left**) and Connolly surface (**right**) of the important growth plane of the ADN/PDO. (**a**) (1 0 0) growth plane (**b**) (0 1 1) growth plane (**c**) (1 1 0) growth plane (**d**) (1 1 −1) growth plane (**e**) (2 0 −2) growth plane.

In the modified attachment energy (MAE) model, S was used to characterize the surface roughness of the (h k l) crystal plane [22]. The larger the value of S, the rougher the surface of the corresponding crystal plane, and the more conducive to the adsorption of solvents [18,23]. It can be seen that the values of S corresponding to (1 0 0), (0 1 1), (1 1 0), (1 1 −1), and (2 0 −2) crystal planes were 1.223, 1.513, 1.359, 1.389, and 1.836, respectively. The packing patterns of five crystal planes of the ADN/PDO and the corresponding Connolly surfaces are shown in Figure 3. Based on Figure 3, more chemical groups were exposed in the (0 1 1) and (2 0 −2) crystal planes compared with the other three crystal planes. It can be seen from Figure 3b,e that the (0 1 1) crystal plane was mainly exposed to the PDO and the cations of the ADN, and the (2 0 −2) crystal plane was exposed to both the cations and anions of the ADN and PDO. This results in the (2 0 −2) crystal plane were more bumpy and rougher than the (0 1 1) crystal plane. The (2 0 −2) plane was the roughest and had more adsorption sites. It was conducive to absorbing the molecule of solvent. This had a certain hindering effect on the growth of the crystal plane.

### 2.3. Morphology of the AND/PDO in Solvent

#### 2.3.1. Binding Energy

When a crystal grows in a solution, the solute molecules first diffuse to the crystal surface through diffusion, then overcome the desorption energy barrier of the solvent, and finally adsorb to the crystal surface to complete the crystal growth [23,24]. Therefore, the crystal morphology can be significantly affected by the adsorption of solvents on the crystal surface. The greater the binding energy between solvent and crystal plane, the stronger the binding effect, the greater the desorption energy barrier that the solute needs to overcome, and the stronger the inhibitory effect of the solvent on the growth of crystal plane. The formula for calculating the binding energy is

(1) $$E_{bind}=-E_{int}=-(E_{tot}-E_{cry}-E_{sol})$$

where *E_bind_*, *E_int_* are the binding energy and interaction energy between the crystal plane and the solvent, respectively. *E_tot_* is the total energy of the mixed system of crystal plane and solvent, *E_cry_* is the energy of the crystal plane in the model, *E_sol_* is the energy of the solvent in the model. The calculation results are shown in Table 3.

**Figure 4:**
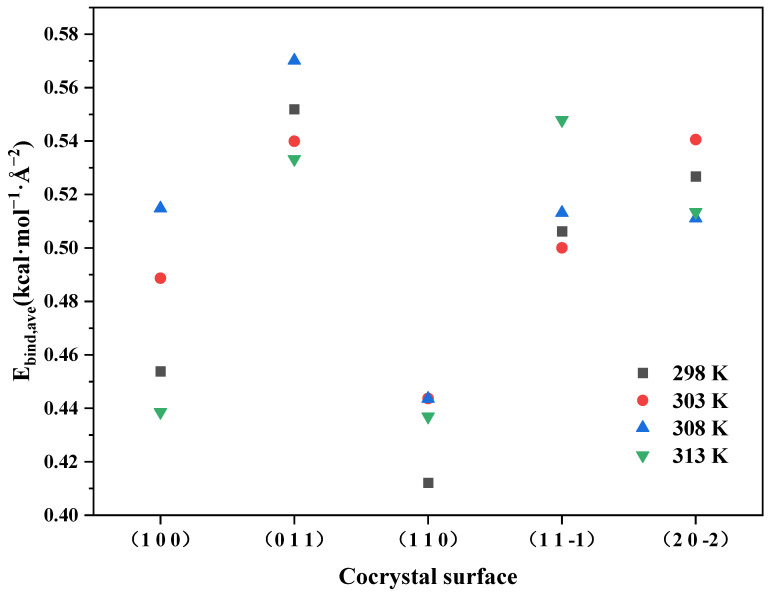
Binding energy between the ADN/PDO and ethanol at different temperatures.

According to Table 3, it can be seen that the binding energy between the ADN/PDO cocrystal and the ethanol solvent were positive on all important growth crystal planes, indicating that the interaction between the solute and the solvent was dominated by attraction. The variations of the binding energy between the ADN/PDO and the ethanol on each important crystal face of the ADN/PDO cocrystal are shown in Figure 4, which were obtained under different temperatures. The order of binding energy between five crystal planes of the ADN/PDO cocrystal and ethanol was (0 1 1) > (1 1 −1) > (2 0 −2) > (1 1 0) > (1 0 0). It can be revealed that the binding effect between the ethanol and the ADN/PDO was the strongest on the (0 1 1) crystal plane. Meanwhile, ethanol had the strongest inhibitory effect on the growth of this crystal plane. It can be seen from Figure 4 that the binding energy between the ADN/PDO crystal plane and ethanol first increased and then decreased as the temperature increased. Its value was always positive, indicating that the process of ethanol adsorption onto the ADN/PDO crystal plane is an exothermic process.

#### 2.3.2. Radial Distribution Function

The radial distribution function (RDF) is often adopted to analyze non-bonding interactions between atomic pairs. It represents the probability of finding atom B at a distance r from atom A. In general, non-bonding interactions between atomic pairs include hydrogen bonding (less than 3.1 Å), van der Waals forces (3.1–5.0 Å), and electrostatic forces (greater than 5.0 Å).

**Figure 5:**
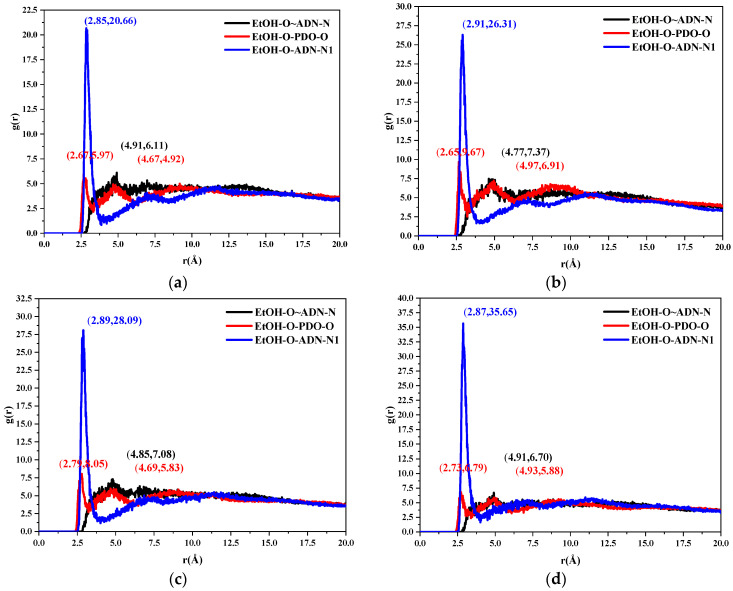
Radial distribution functions between the oxygen atoms in the ethanol and the oxygen atoms in the PDO, the nitrogen atoms in the ADN in the (0 1 1) plane at different temperatures. (**a**) 298 K (**b**) 303 K (**c**) 308 K (**d**) 313 K.

Taking the (0 1 1) crystal plane as an example, the RDF results are shown in Figure 5. The black, red, and blue curves corresponded to the RDF between the oxygen atom in ethanol (EtOH-O) and the nitrogen atom connected to two nitro groups in the ADN anion (ADN-N), the RDF between the EtOH-O and the oxygen atom in the PDO (PDO-O), and the RDF between the EtOH-O and the nitrogen atom in the ADN cations (ADN-N1), respectively. The coordinates of the local maximum value of each curve are given in the Figure 5. Based on Figure 5a, the RDF of the EtOH-O and ADN-N1 had the first peak at r = 2.85 Å at 298 K, indicating that there was a hydrogen bond between the ethanol and ADN cations. The peak of the black curve appeared at the position of r = 4.91 Å, indicating that there was a van der Waals interaction between the ethanol and the ADN anions. There were two peaks in the radial distribution function between the EtOH-O and PDO-O, which appeared at r = 2.67 and 4.67 Å respectively. This revealed the presence of hydrogen bonds and van der Waals interactions between the ADN and the PDO.

Similarly, when the temperatures were 303, 308, and 313 K, there were hydrogen bonds and van der Waals interactions between the ethanol and the PDO. There were hydrogen bonds and van der Waals interactions between the ethanol and the cation and anion of the ADN, respectively. As the temperature increased, the number of local maximum values of the three RDFs remained unchanged, while the abscissa of the local maximum point moved to the right as a whole. This revealed that the increase in temperature led to the change of the hydrogen bond and van der Waals interaction between the ethanol and the ADN, PDO, which first increased and then decreased. It was consistent with the binding energy results in Figure 4.

#### 2.3.3. Diffusion Coefficients

**Figure 6:**
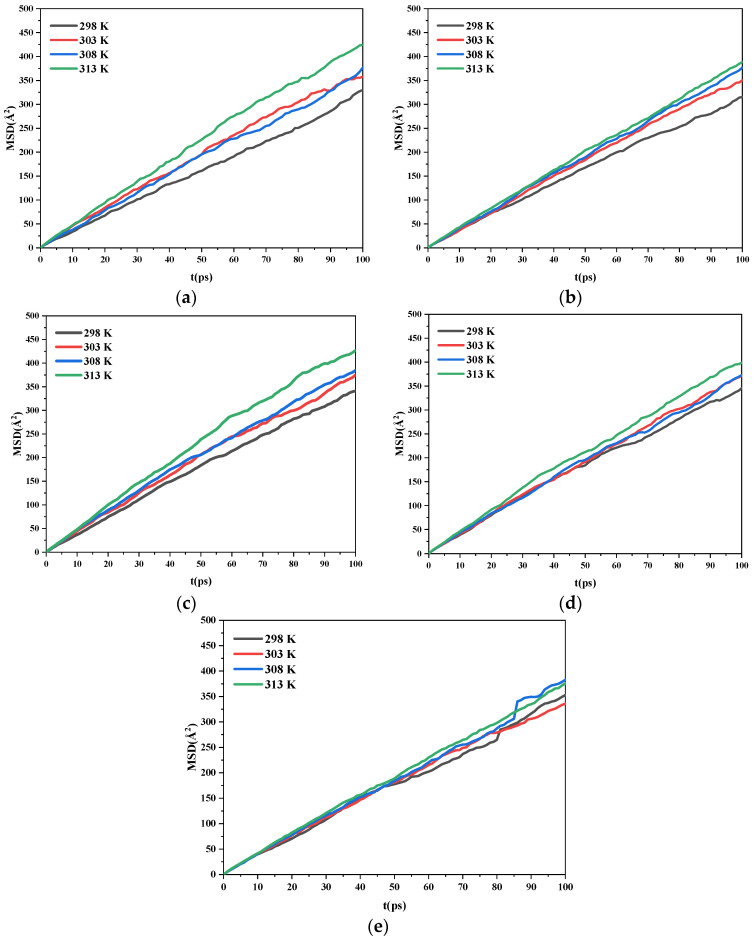
Mean square displacement curves of the solvent in the ADN/PDO-EtOH layered model at different temperatures. (**a**) (1 0 0) (**b**) (0 1 1) (**c**) (1 1 0) (**d**) (1 1 −1) (**e**) (2 0 −2).

Generally, the diffusion of a solvent is greatly affected by temperature. The diffusion of a solvent on the crystal surface may cause it to adsorb on the crystal surface. Absorption of solute molecules to the crystal surface is blocked by this process. The growth of the corresponding crystal planes is also inhibited. To reveal the effect of temperature on the diffusion of ethanol molecules on the AND/PDO crystal plane, the calculated trajectories were processed, and the corresponding mean square displacement curves were calculated, which is given in Figure 6.

According to Einstein’s law of diffusion, the formula for calculating the diffusion coefficient is

(2) $$\underset{t\to \infty }{\lim}\langle {\left|\vec{r}(t)-\vec{r}(0)\right|}^{2}\rangle =\underset{t\to \infty }{\lim}\mathrm{MSD}=6Dt$$

where *D* is the diffusion coefficient. Therefore, the diffusion coefficient is one-sixth of the slope of the mean square displacement curve.

The diffusion coefficients of the ethanol on each crystal surface at different temperatures are given in Table 4. Taking the (0 1 1) surface as an example, when the temperature was 298 K, the diffusion coefficient of the ethanol in the system was 0.52 × 10^−8^ m^2^·s^−1^. When the temperature rose to 313 K, the diffusion coefficient of the system was 0.64 × 10^−8^ m^2^·s^−1^. As the temperature increased, the diffusion coefficient of the ethanol under the same crystal plane increased gradually. At the same temperature, the largest diffusion coefficient of the ethanol in the layered model was obtained under the (1 1 0) crystal plane. However, the final morphology of the crystals in the solvent was determined by the diffusion of the solute and solvent and the competitive adsorption of the solute and solvent on the crystal surface.

#### 2.3.4. Morphology Analysis

**Figure 7:**
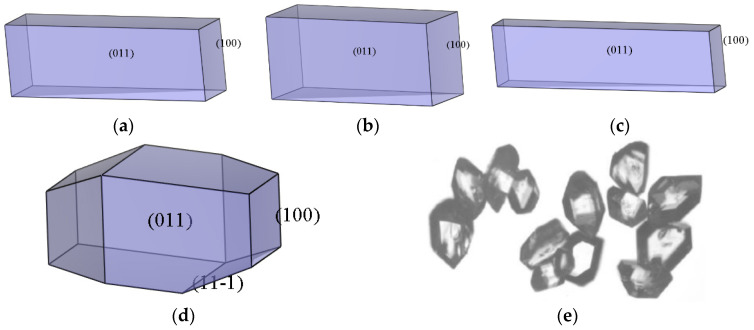
Morphology of the ADN/PDO cocrystal in ethanol and the ADN/PDO cocrystal morphology at different temperatures. (**a**) 298 K (**b**) 303 K (**c**) 308 K (**d**) 313 K (**e**) experiment.

According to the modified AE model, the corrected attachment energy and aspect ratio between the crystal plane of the ADN/PDO cocrystal and ethanol are shown in Table 5, which was calculated under different temperatures. The calculated morphology of the ADN/PDO cocrystal in ethanol at different temperatures and the corresponding experimental crystal morphology of the ADN/PDO cocrystal [25] are shown in Figure 7. Based on Table 5, it can be seen that the (0 1 1) crystal surface of the ADN/PDO cocrystal had a greater interaction with the ethanol than the (1 0 0) plane at the four temperatures (298, 303, 308, and 313 K). This indicated that the ethanol had a strong inhibitory effect on the growth of the ADN/PDO (0 1 1) crystal surface.

According to the results of S on each crystal plane of the ADN/PDO cocrystal, the (2 0 −2) crystal plane was the roughest, which was the most favorable for the adsorption of solvents. However, the binding energy between the solvent and the (2 0 −2) crystal plane was weak, and the diffusion coefficient of ethanol on the (2 0 −2) plane was small. Under the comprehensive influence of various factors, the relative growth rate of the (2 0 −2) plane was large, which made the (2 0 −2) crystal surface disappear eventually during the growth process. Similarly, the roughness of the (0 1 1) surface and the binding energy between explosives and ethanol were large. This gave the (0 1 1) surface the largest area ratio in the ethanol solvent.

When the temperature was lower than 308 K, the morphology of the ADN/PDO cocrystal in the ethanol solvent was a quadrangular prism. The three crystal planes (1 1 0), (1 1 −1), and (2 0 −2) eventually disappeared during the growth process owing to the high relative growth rate. When the temperature increased from 298 to 308K, the (0 1 1) surface area ratio of the ADN/PDO cocrystal was greater than 80%, and the aspect ratios of the ADN/PDO cocrystal were 3.324, 2.653, and 4.090, respectively. When the temperature was 313 K, the morphology of the ADN/PDO cocrystal was irregular prism, and the aspect ratio was 2.353. The ADN/PDO cocrystal morphology calculated by the modified AE model was the closest to the experimental one at 313 K. The presented modeling and simulations can also be applied for the prediction of the drug crystal topologies in a pharmaceutical application employing drug delivery carriers [26,27,28].

## 3. Modeling and Simulation

### 3.1. Modified Attachment Energy Model

The Attachment Energy Model (AE model) was developed by Hartman and Bennema [25,29] on the basis of the Periodic Bond Chain (PBC) theory. In the AE model, the relative growth rate of each crystal plane of the crystal is proportional to the absolute value of the crystal plane attachment energy.

(3) $$R_{hkl}\propto \left|E_{att}\right|$$

where *R_hkl_* is the relative growth rate, and *E_att_* is the attachment energy of the crystal plane, kcal·mol^−1^. The attachment energy (*E_att_*) is defined as the energy released by a wafer with a thickness of d_hkl_ attached to the (h k l) crystal plane. The formula is

(4) $$E_{att}=E_{latt}-E_{slice}$$

where *E_att_*, *E_latt_*, *E_slice_* are the attachment energy, lattice energy of crystal, and the energy of growth slice, respectively.

By calculating the attachment energy of the crystal face, the habit of the crystal can be predicted with the help of the AE model. However, the external environment of crystal growth is not considered by the AE model. It is difficult to accurately reveal the actual growth process of crystals in solution [30,31]. Therefore, the AE model should be corrected, and the modified formula for calculating the attachment energy is

(5) $$E_{att}^{\prime }=E_{att}-S\cdot E_{s}$$

where *S* is used to describe the surface characteristics and is defined as $S=\frac{A_{acc}}{A_{hkl}}$.

Among them, *A_acc_* is the accessible area of solvent on the crystal plane unit (h k l), *A_hkl_* is the cross-sectional area of the crystal plane unit (h k l). *E_s_* represents the effect of the solvent on the crystal plane growth, which is defined as

(6) $$E_{s}=E_{\mathrm{int}}\cdot \frac{A_{hkl}}{A_{box}}$$

where *A_box_* and *E_int_* are the cross-sectional area of the simulation box and the interaction energy between the crystal layer and the solvent, respectively.

*E_int_* is defined as

(7) $$E_{\mathrm{int}}=E_{tot}-(E_{cry}+E_{sol})$$

where *E_tot_*, *E_cry_*, *E_sol_* are the total energy of the mixed system of crystal plane and solvent, the energy of crystal plane in the calculation model, and the energy of the solvent in this model, respectively. Meanwhile, under solvent conditions, the relative growth rate of the crystal is proportional to the absolute value of the corrected attachment energy.

(8) $$R_{hkl}^{\prime }\propto \left|E_{hkl}^{\prime }\right|$$

### 3.2. Computational Methods

**Figure 8:**
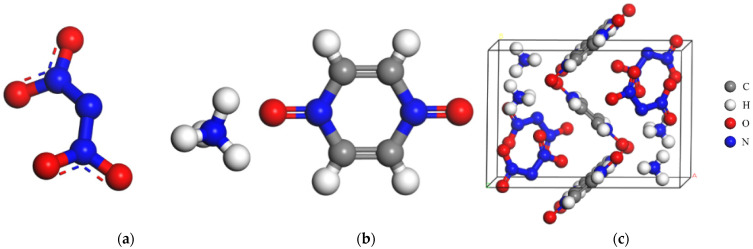
ADN molecular structure, PDO molecular structure, and unit cell structure of the ADN/PDO cocrystal. (**a**) ADN molecular structure (**b**) PDO molecular structure (**c**) Unit cell structure.

The initial unit cell for the ADN/PDO cocrystal was obtained from the Cambridge Crystallographic Data Centre (CCDC) [14]. The initial unit cell parameters of the ADN/PDO cocrystal were a = 11.592 Å, b = 8.188 Å, c = 7.227 Å, α = γ = 90°, β = 101.236°, and the space group was P21/c, belonging to the monoclinic crystal system. The molecular structure of the ADN and PDO and the unit cell structure of the ADN/PDO cocrystal are shown in Figure 8. In Figure 8, the gray, white, red, and blue balls correspond to carbon, hydrogen, oxygen, and nitrogen atoms, respectively.

**Figure 9:**
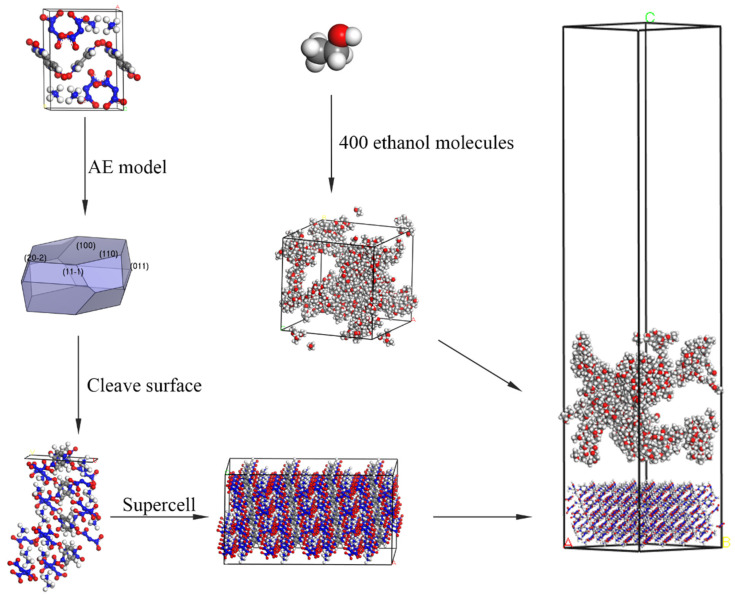
Schematic diagram of the flow chart of calculation model construction.

The flowchart of the calculation model is given in Figure 9. At first, the stable structure of the ADN/PDO unit cell was obtained after geometry optimization. The Smart optimization algorithm was used, which is the built-in algorithm in Materials Studio 2020. The Ewald summation method was selected as the electrostatic force summation method, and the precision was set to 0.001 kcal·mol^−1^. The atom-based method was selected as the van der Waals force summation method, and the cut-off radius was set to 12.5 Å. The morphology of the AND/PDO in vacuum was predicted with the help of the AE model. The important growth planes under vacuum conditions were determined. Then, the important crystal planes of the ADN/PDO were cut to expand it into a 4 × 4 × 4 supercell structure. A solvent layer containing 400 ethanol molecules was constructed using the Amorphous Cell module. The Build Layers function was used to construct an “explosive/solvent” double-layer model with a 50 Å vacuum layer above the solvent layer.

The model was optimized using the Forcite module of Materials Studio 2020, followed by molecular dynamics calculations at specified temperatures (298, 303, 308, and 313 K). When molecular dynamics calculations were performed, the ensemble and the simulation time were set as NVT and 500 ps, respectively. A total of 500,000 calculation steps were performed, and the timestep was set as 1 fs. The Andersen method was adopted to control the temperature through the whole simulation process. The initial velocity of the particle was randomly assigned according to a Gaussian distribution. Finally, the homemade script was used to calculate the interaction energy between the ADN/PDO explosive and ethanol, the radial distribution function, the mean square displacement, and the mass density distribution based on the stable part of the calculated trajectory.

## 4. Conclusions

In summary, the modified attachment energy (MAE) model was adopted to investigate the morphology of the ADN/PDO cocrystal in vacuum and ethanol at different temperatures (298, 303, 308, and 313 K). The CVFF forcefield and Mulliken charges were used to perform the molecular dynamics simulation. The main conclusions of this work are as follows:

(1) The growth morphology of the ADN/PDO is hexagonal prism in vacuum, and the five main crystal surfaces are (1 0 0), (0 1 1), (1 1 0), (1 1 −1), and (2 0 −2). Among them, the (1 0 0) surface has the largest exposed area, accounting for 40.744%.
(2) The binding energy between the ADN/PDO cocrystal and ethanol solvent is positive on all important growth planes. The order of binding energy is (0 1 1) > (1 1 −1) > (2 0 −2) > (1 1 0) > (1 0 0). The binding effect between the ethanol and ADN/PDO is strongest on the (0 1 1) crystal plane.
(3) The radial distribution function analysis of the (0 1 1) crystal plane showed that there are hydrogen bonds between the ethanol and ADN cations, van der Waals interactions with the ADN anions, and hydrogen bonds and van der Waals interactions with the PDO at the same time. In the (0 1 1) crystal plane, the value of S is 1.513, which indicates that this surface has a large roughness. This is more conducive to the adsorption of ethanol molecules.
(4) As the temperature increases, the diffusion coefficient of the ethanol under the same crystal plane increases gradually. Meanwhile, the morphology analysis indicated that increasing the temperature is beneficial to reducing the aspect ratio of the crystal. This is conducive to the reduction of explosive sensitivity.

## Figures and Tables

**Table 1:** ADN/PDO unit cell parameters and relative errors optimized by five force fields.

Parameters	Exp	COMPASSIII	PCFF	CVFF	Universal	Dreiding
a/Å	11.592	10.946	11.533	11.626	11.819	11.347
b/Å	8.188	8.143	9.167	8.504	8.472	7.960
c/Å	7.227	6.832	6.946	7.217	7.687	7.696
α/°	90.000	90.000	90.000	90.000	90.000	90.000
β/°	101.236	99.546	93.744	101.050	100.974	100.628
γ/°	90.000	90.000	90.000	90.000	90.000	90.000
RE_a_	0.00%	−5.57%	−0.51%	0.29%	1.96%	−2.12%
RE_b_	0.00%	−0.54%	11.96%	3.86%	3.47%	−2.78%
RE_c_	0.00%	−5.47%	−3.89%	−0.14%	6.36%	6.49%
RE_α_	0.00%	0.00%	0.00%	0.00%	0.00%	0.00%
RE_β_	0.00%	−1.67%	−7.40%	−0.18%	−0.26%	−0.60%
RE_γ_	0.00%	0.00%	0.00%	0.00%	0.00%	0.00%

Note: RE is the relative error.

**Table 2:** The crystal habits parameters of the ADN/PDO cocrystal under vacuum (attachment energy model).

(h k l)	d_hkl_	Surface Area/Å^2^	E_att (total)_/(kcal·mol^−1^)	Total Facet Area/%	Aspect Ratio
(1 0 0)	11.411	61.370	−61.570	40.744	2.569
(0 1 1)	5.442	128.669	−117.417	26.208	
(1 1 0)	6.818	102.703	−109.348	18.177	
(1 1 −1)	5.220	134.151	−119.789	14.450	
(2 0 −2)	3.306	105.900	−148.904	0.422	

**Table 3:** Energy details of the layered model for each crystal face of ADN/PDO-EtOH at different temperatures.

T/K	(h k l)	E_tot_/ (kcal·mol^−1^)	E_sol_/ (kcal·mol^−1^)	E_cry_/ (kcal·mol^−1^)	E_bind_/ (kcal·mol^−1^)
298	(1 0 0)	−31,481.17	−977.69	−30,057.88	445.60
	(0 1 1)	−30,628.73	−1821.46	−27,671.08	1136.19
	(1 1 0)	−34,837.67	−1936.03	−32,224.39	677.25
	(1 1 −1)	−36,757.43	−1570.34	−34,100.57	1086.52
	(2 0 −2)	−13,735.02	−1740.63	−11,101.82	892.57
303	(1 0 0)	−31,274.01	−817.80	−29,976.35	479.86
	(0 1 1)	−30,552.83	−1790.10	−27,651.20	1111.53
	(1 1 0)	−34,632.60	−1770.36	−32,133.13	729.11
	(1 1 −1)	−36,837.35	−1703.37	−34,060.68	1073.30
	(2 0 −2)	−13,690.91	−1658.16	−11,116.84	915.91
308	(1 0 0)	−31,210.31	−829.50	−29,875.30	505.50
	(0 1 1)	−30,341.76	−1626.49	−27,541.56	1173.72
	(1 1 0)	−34,523.77	−1706.96	−32,087.83	728.98
	(1 1 −1)	−36,633.24	−1583.95	−33,947.88	1101.40
	(2 0 −2)	−13,497.78	−1561.47	−11,070.35	865.96
313	(1 0 0)	−31,010.92	−714.65	−29,865.60	430.68
	(0 1 1)	−30,239.24	−1560.73	−27,580.70	1097.81
	(1 1 0)	−34,250.38	−1550.09	−31,982.37	717.92
	(1 1 −1)	−36,502.03	−1486.06	−33,840.10	1175.87
	(2 0 −2)	−13,428.79	−1492.03	−11,066.84	869.92

**Table 4:** Diffusion coefficient of the solvent on each crystal face at different temperatures.

(h k l)	D/(×10^−8^ m^2^·s^−1^)			
	298 K	303 K	308 K	313 K
(1 0 0)	0.53	0.61	0.60	0.71
(0 1 1)	0.52	0.59	0.62	0.64
(1 1 0)	0.57	0.61	0.63	0.73
(1 1 −1)	0.56	0.61	0.60	0.66
(2 0 −2)	0.57	0.56	0.62	0.61

**Table 5:** Modified attachment energies and related parameters of the ADN/PDO cocrystals in ethanol at different temperatures.

T/K	(h k l)	E_int_/ (kcal·mol^−1^)	E_s_/ (kcal·mol^−1^)	E_att_′/ (kcal·mol^−1^)	Total Facet Area/%	Aspect Ratio
298	(1 0 0)	−445.60	−27.85	−27.51	15.37	3.324
	(0 1 1)	−1136.19	−71.01	−10.00	84.63	
	(1 1 0)	−677.25	−42.33	−51.81	-	
	(1 1 −1)	−1086.52	−67.91	−25.46	-	
	(2 0 −2)	−892.57	−55.79	−46.46	-	
303	(1 0 0)	−479.86	−29.99	−24.90	19.85	2.653
	(0 1 1)	−1111.54	−69.47	−12.33	80.15	
	(1 1 0)	−729.11	−45.57	−47.40	-	
	(1 1 −1)	−1073.30	−67.08	−26.61	-	
	(2 0 −2)	−915.91	−57.24	−43.78	-	
308	(1 0 0)	−505.50	−31.59	−22.94	12.33	4.090
	(0 1 1)	−1173.72	−73.36	−6.45	87.67	
	(1 1 0)	−728.98	−45.56	−47.41	-	
	(1 1 −1)	−1101.40	−68.84	−24.17	-	
	(2 0 −2)	−865.96	−54.12	−49.52	-	
313	(1 0 0)	−430.68	−26.92	−28.65	10.70	2.353
	(0 1 1)	−1097.81	−68.61	−13.63	61.50	
	(1 1 0)	−717.92	−44.87	−48.35	-	
	(1 1 −1)	−1175.87	−73.49	−17.71	27.80	
	(2 0 −2)	−869.92	−54.37	−49.06	-	

## Data Availability

Not applicable.
